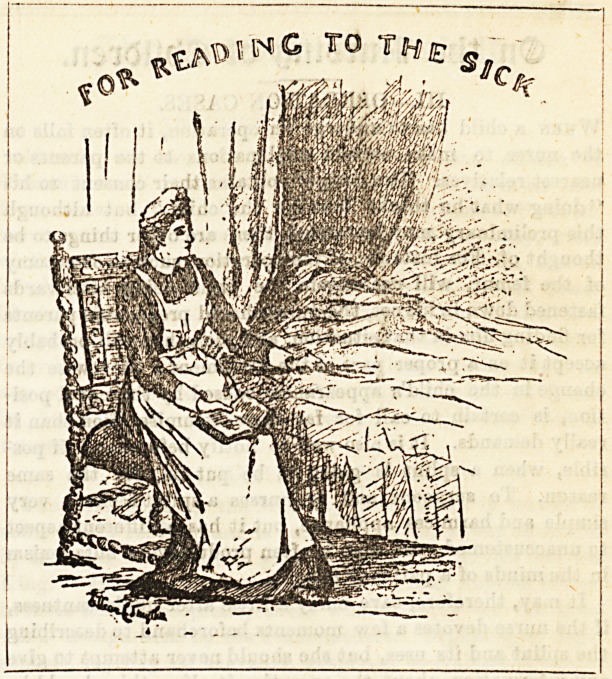# The Hospital Nursing Supplement

**Published:** 1892-02-27

**Authors:** 


### "The Hospital" Nursing Mirror.

Being the Extra Nursing Supplement of "The Hospital" Newspaper.

Contributions for this Supplement should be addressed to the Editor, The Hospital, 140, Strand, London, W.C., and should have the word "Nursing" plainly written in left-hand top corner of the envelope.

### En Passant.

The L. O. S. Examination.—The following candidates from St. John's Maternity Home, Battersea, have successfully passed the examination of the London Obstetrical Society held in January: Sister Rosalie, Sister Alma, Sister Lois, and Sister Margaretta, of the Community of St. Peter, Kilburn; Emily Constance Gobat, Mary Denning, Mary Ann Skinner, Anne Jane Henry, Mary Frances Sulston.

Southend Victoria Hospital.—This small institution, after a rather troubled start in life, seems to have settled down now to do good and quiet work. The number of patients admitted last year was 56, as against 46 in 1890; the cases were mostly of a severe type, and six deaths occurred. The patients are supposed to make a small weekly payment, and two regular paying cases have been received. Last November Miss C. Gunn was elected Matron of the hospital, and she seems to be giving every satisfaction to the Committee.

The Nurse Dolls.—The bazaar held at Burslem has proved a great success; it was first started by Miss Moore and the nurses of the Haywood Hospital to raise funds for the annual Christmas tree, and there was such a ready response from the townspeople that this year Miss Moore decided to try and start a district nurse through its means. In spite of influenza and bad weather about £800 was realised, and we heartily congratulate all concerned. The nurse-dolls were on show, and as thanks for their loan Miss Moore has sent £1 for the nurses' bed. The dolls are not going to Bath until the autumn, so that their next public appearance will probably be at Bradford in May.

Manchester Nurses.—The 26th annual report of the Manchester and Salford Sick Poor and Private Nursing Institution is a bulky and interesting pamphlet. There was profit of £404 on the private nurses' work last year which was handed over to help maintain the district nurses, a form of charity against which we have frequently inveighed. It seems that one-third of the whole of the voluntary contributions comes from nine persons, another proof of Mr. Conrad Theis' contention, that it is not the many who give. The report gives a map of Manchester showing the various districts in which the nurses work, also it gives the rules for nurses and for employers. It is in every way a credit to the Hon. Secretary.

York Home for Nurses.—A very good report was submitted at the annual meeting of this Home held on February 15th. The staff employed during the year consisted of the Sister-in-charge, two sisters working amongst the sick poor, 53 nurses, 2 district nurses, and 20 probationers. The number of nurses has been steadily increasing. The reports received of the nursing have been very good, with a few exceptions. A resolution was passed that a bonus should occasionally be given to nurses who had served the Home with credit for five years and upwards. Seven of the staff had, during the year, passed on to good appointments. The Council expressed their gratitude to the Sisterhood of the Holy Cross for the excellent management of the Home.

Short Items.—The Commissioners in Lunacy have ordered an infirmary to hold 102 beds to be built for Banstead Asylum; let us hope they will insist on a trained nurse being put in charge.—The late Sir Morell Mackenzie gave Miss Mackey and Sister Edith silver crosses in recognition of their aid in his experiments with Koch's fluid.—Miss Harbord has opened an Institute for Trained Nurses at Margate.—Miss Parry's lectures in Essex are being fully reported in the local papers.—The Duchess of Bedford lately distributed a lot of St. John's certificates to the ladies and policemen of Bedford.—The Rural Branch of the Q.V.J.N.I. is now training 22 probationers, and Mrs. Malleson appeals for funds.—The Egham District Nurse paid 2,765 visits last year.—A nurse is needed in Elgin; let us hope the rich will see to it.—Mr. Gordon Elliot has started a nursing institute at Acton.—Dr. Broadbent has been gazetted Physician in Ordinary to the Prince of Wales. —Mrs. Barrie has been appointed Secretary of the fund for providing a trained nurse for Dumfries; £113 has been subscribed.

The Nurse and the Master.—The lines of the workhouse nurse do not often fall in pleasant places, and by no means the least difficulty of her position is generally the attitude of the Master and Matron of the union. The class of "respectable couples" from which the Master and Matron are chosen is slightly lower than that from which the nurse is probably taken, nevertheless the Master is put in authority over the nurse, and then trouble begins. The Master is absolutely ignorant of nursing, he naturally dislikes the extra trouble necessary to getting the infirmary into order, and he regards the nurse as an uppish young woman. Five nurses in succession have thrown up their posts at Hayfield Union, and the Guardians have now taken the inadequate step of admonishing the Master and Mistress, and requesting them to treat the new nurse with more respect. The only thorough way of stopping quarrels of this sort is to make the nurse quite independent of the Master, and responsible to the Medical Officer.

The Hampshire Institute.—Surgeon-General Dr. Maclean lately presided at the annual meeting of this Institute and read the report for last year. It stated that the Institute had supplied trained nurses to 110 families, 70 cases were declined, but in every case where possible an outside nurse was provided. The fees earned during the year amounted to £620 12s., being £9 19s. in excess of the previous year. The housekeeping charges amounted to £173 13s. 8½d., a decrease on the previous year of £19 4s. 11½d. There was no case of serious illness among the staff during the year. The staff consisted of 14 trained nurses, but not enough to meet the wants of the public. The Committee had, by persistent advertising, endeavoured to increase the number without success, presumably on account of the great demand already mentioned. Two probationers had completed their training, and were now at work. Four probationers were now in course of training, and the effective staff on January 31st consisted of sixteen trained nurses. Dr. Trend, in seconding the adoption of the report, spoke very highly of the skill and general tone of the nurses. A vote of thanks to the Matron for her services was carried unanimously.

### Lectures on Surgical Ward Work and Nursing.

By Alexander Miles, M.D. (Edin.), F.R.C.S.E.

### Lecture XLVI.—CONCLUSION.

Of Enema Syringes perhaps the simplest and the best is the ordinary Higginson's Syringe (Fig. 6a); others are more complicated, without any corresponding advantage. Some

Fig. 1. Fig. 2.

act on the syphon principle and are very useful. For the adminstration of nutrient enemata the instrument most convenient is a simple indiarubber bag with nozzle. Other

Fig 3.

Fig. 4.

Fig. 5.

Fig. 7.

instruments used in operations on the rectum, such as hooks, scissors, directors, and so on, do not require special mention.

Some Instruments used in the Larynx.—(1) Laryngoscope :—This apparatus consists of several distinct pieces. (a) The reflector, which is fitted to the operator's head either by means of a forehead band, or on a spectacle frame-work. This is to cast a bright light into the patient's mouth. (b) The laryngeal mirror, which is a small round mirror fixed at an obtuse angle to a long slender stalk, and in which the observer sees the reflection of the parts in the larynx. There are six such mirrors of different sizes, all fitting into one

Fig. 6a.

handle. (c) For diagnostic, as well as therapeutic purposes, probes and brushes may be fitted to the handle (Fig. 1). (2) Laryngeal Syringe is used to inject substances such as menthol into the larynx (Fig. 2); and (3) Laryngeal Insufflators are employed when it is desired to apply any powder to the parts (Fig. 3). (4) Laryngeal Forceps, such as those of Mackenzie, are employed in various operations on the larynx. Some open laterally, others antero-posteriorly (Fig. 4).

Fig. 8.

Fig. 9.

Fig. 10.

Tracheotomy Instruments.—The operation of Tracheotomy may be performed with very few instruments, and the less specialised these are the better. Of varieties of Tracheotomy tubes there is almost no end. One of the commonest is that figured (Fig. 5).

Ear Instruments.—(1) Specula, are short conical tubes of different sizes, and are used when it is desired to examine the condition of the outer ear or tympanic membrane

(Fig. 7). Some are mounted on handles and have two blades which may be separated, thus widening the canal.

(2) Mirrors or reflectors are used to illuminate the parts, as in the larynx.

(3) Aural Forceps. The most useful form of these act with a spring, and have an obtuse angle in them so that the observer's hand is out of the line of vision (Fig. 8), others act with compound joints. Many other contrivances have been introduced with which to remove foreign bodies, or new growths from the ear, such as hooks, scoops, snares, and so on.

(4) The Eustachian catheter (Fig. 9) is used in conjunction with Politzer's bag and the Otoscope (Fig. 10) both in the diagnosis and treatment of aural affections.

Nasal Instruments.—(1) Specula. Here again a great many forms of instruments for diagnostic examination have

Fig. 11.

Fig. 12.

been introduced, *e.g.* (*a*) Frankels' Nasal Speculum; (Fig.11) that by Lennox Browne, that of Thudichum, and many others.

(2) Beloque's Sound is used in the plugging of the posterior nares. It consists of a hollow curved stem containing a piece of watch spring. To the latter a thread is fixed and the sound being passed along the inferior meatus of the nose the spring is released by a screw at the end, and it curls into the mouth, carrying the thread with it. The thread is seized and the sound withdrawn. A suitable plug now being attached to the string is pulled into position and retained there (Fig. 12).

(3) Polypus Forceps are strong bladed forceps, of the same pattern as dressing forceps, but the blades are longer and serrated throughout on their inner aspect.

The illustrations are used by kind permission of Messrs. Maw, Son, and Thompson and Messrs. Arnold and Sons.

### Appointment.

[It is requested that successful candidates will send a copy of their applications and testimonials, with date of election, to The Editor, The Lodge, Porchester Square, W.]

Ledbury Cottage Hospital.—Miss Emily Steel has been appointed Matron of this hospital; she trained at Westminster, and after taking her certificate took charge of wards in provincial hospitals, till in 1890 she was appointed Matron of the Brentwood Cottage Hospital, a post she vacated last June.

### DISCIPLINE.

All young folks look forward to the time when they shall leave school and be free from tutors and governors. Some intend to take their pleasure only, others to work and make a fortune, others to plunge into folly and dissipation, but all sooner or later find themselves disappointed. The fact is we ought never to cease from learning, or free ourselves from all restraint, but put ourselves into the ranks of Christ's disciples, and learn of and be governed by Him, otherwise we shall be drawn into the wiles of Satan and become his scholars. "But I hate wickedness," some of us will say, "and never mean to take sides with the enemies of God. I only want to have my own way and do as I like." Very well, that is just the attitude of the mind which requires discipline, and our Heavenly Father in His true and great love sends us trials and losses, sickness and suffering, till we learn to say "what Thou doest is well." It takes some of us a long time to get this lesson by heart, for we do not see why such a task is set us, and think our afflictions are punishments and signs of God's anger. But it is not so. "The Lord loveth whom he chasteneth," and though He sometimes allows the just consequences of our sin or folly to fall heavily on us, yet oftenest the correction is only given lightly, as a warning that by nature we cannot keep straight, but must be led by the teaching of the Holy Spirit, who will guide us into all truth.

Again, we must remember that this life is not the end of our existence, but a preparatory school only, a place where we learn to fit ourselves for the work or rest of another life, a life of such bliss and happiness, that eye hath not seen, nor ear heard, neither have entered into the heart of man, the things which God hath prepared for them that love Him, and where there will be no more death, neither sorrow, nor crying, neither shall there be any more pain, for the former life will have passed away.

Let us, then, take our pain and sorrow, the gentle chidings of a loving Father, with submission, and try to fit ourselves for the glorious future which awaits those who love God and keep His Commandments. Not thinking, as many foolish people do, that we shall be unhappy, for

Why should we fear, youth's cup of joy,
If pure, would sparkle less?
Why should the cup the sooner cloy
Which God hath deigned to bless?

The poet Tennyson says to manhood:—

"To live by law, acting the law we live by,
And, because right is right, to follow right
Is wisdom."

We Christians can add, and happiness also, for ours is the perfect law of liberty, and the Master we learn of is meek and lowly, and we shall find rest unto our souls, for His yoke is easy and His burden is light.

### On the Nursing of Children.

### III.—OPERATION CASES.

WHEN a child has to undergo an operation, it often falls on the nurse to make certain explanations to the parents or nearest relatives. The surgeon obtains their consent to his "doing what he thinks best for the child," but although this preliminary may be settled, there are other things to be thought of. For instance, if the operation, such as osteotomy of the femur, will necessitate the child being afterwards fastened down in his cot, the nurse should prepare his parents for finding him in that situation, and then they will probably accept it as a proper part of the treatment; otherwise the change in the child's appearance, caused by the novel position, is certain to call for far more commiseration than it really demands. It is also well to notify beforehand, if possible, when a splint is going to be put on, for the same reason. To surgeons and to nurses a splint looks a very simple and harmless appliance, but it has a different aspect to unaccustomed eyes, and is often productive of antagonism in the minds of a patient's friends.

It may, therefore, save many a little after unpleasantness, if the nurse devotes a few moments beforehand to describing the splint and its uses, but she should never attempt to give any information about the operation itself; this should be left entirely to the surgeon, who is always willing to tell as much as is necessary.

When an unfortunate child has "been on the table" before, it is impossible to prevent his knowing something of what is coming; but when it is a first operation, it is as easy as it is desirable to keep him in ignorance.

He should be specially cared for during the previous hours, and the last meal that he is permitted should be a suitable and sufficient one, of which he should partake in a comfortable and leisurely fashion. It ought to be of a particularly appetising character, so that it bears something of the nature of "a feast" about it.

If we are dealing with a child in hospital, and the operation has to take place in the afternoon, the hour when the others dine, and he does not, is a trying one, and it is well worth taking a little trouble to spare the child the disappointment of seeing others partake of a meal which he alone is denied.

It seems a small matter to mention, but to the little patient it is an all-important one, and it is no uncommon thing for him to announce his return to consciousness after an operation with the words, "Nurse! I want my dinner." Few adults are capable of such a proceeding.

To the child the fear that he has missed his regular meal is far more of a grievance than anyone who has not been present at such a moment could possibly imagine. Of course, this is chiefly evident in dealing with the children of the poor, to whom a regular meat dinner in the ward is a daily and delightful event.

A little girl of five, who, after various conservative operations, was obliged to have her leg amputated at the hip, was an example of this. She had been very bad for a short time, and was still being watched with some degree of anxiety, when she suddenly opened her eyes and demanded her dinner; moreover, being a very determined little person, she declined to be consoled by her nurse's promise of a feast later on. She made so much noise that the kindly house surgeon was summoned, and he being claimed by her as "my own doctor," succeeded, by dint of a great deal of coaxing, and a little scolding and laughter, in prevailing on the wilful little mite to be quiet for awhile, and postpone the discussion of the plate of mince with "taters and gravy" on which she had set her mind.

The special bath and "general polishing up" which a thorough surgical nurse enjoys bestowing on her "case," must be judiciously arranged, so as neither to fatigue nor chill the child. He should then be enveloped in a good blanket, and have warm white socks on, in addition to the night-gown, which should open in front. If it is a very young child, the night-gown may have to be dispensed with at the last, but then there must be an additional blanket or square of new flannel which should be small enough not to impede the surgeon, and large enough to ensure warmth to the tender little body.

The aperient and enema ordered to be given beforehand, should be administered accurately to time, and every care taken to avoid an unpleasant disaster "on the table," which depends greatly on the nurse's good management.

But in pleasant and unpleasant duties alike, she should bear in mind the necessity for not hurrying or worrying her little patient. If she keeps him comfortable, warm, and cheerful up to the last moment, she has done her very best to make and keep him "fit" for the operation.

After this is over, her duties are generally very clearly defined. She has to practise the most implicit obedience to the doctor's orders, and she must be careful to thoroughly comprehend these. It is no use to say, after making a blunder, "I thought he meant so and so," or "I did not like to ask questions, for fear he should think me stupid."

Intelligent questions are never foolish ones, and as a patient's safety and comfort are the first consideration, it is well for a nurse to learn early in her career that her own feelings must take a very secondary position. In fact, a thoroughly earnest woman learns to set all consideration of self so completely in the background, that she becomes quite free from that self-consciousness which is the snare of weaker, vainer natures. After the first few days a case of osteotomy seldom causes any special anxiety, but of course the patient must be kept very quiet, and no one who has not tried it can appreciate the extreme difficulty of keeping a child still; the better the general health and spirits, the harder the task.

However, here again common sense shows us that the child can be reconciled to the absolute rest needed for the leg or legs, and of what good is a nurse who lacks this valuable gift, wrongly called "common" sense? For instance, some one gives the child a charming book of pictures, which is so cumbersome and heavy that he soon instinctively lets the weight of it lie on the mattress, and he gradually shifts himself round to enjoy the illustrations until he lies in a most undesirable curved position. The lightest books and smallest toys are the best for childen in bed. All objects designed to keep them amused and quiet should be placed before them, and they would then be attracted to the bed-table or board which can be fixed in a convenient position. If the child's head may be raised without risk to him, let it be put on firm pillows and avoid, for him, the fatigue of keeping it up without proper support.

*(To be continued.)*

### Presentations.

AT Wigan Infirmary on February 10th the Mayoress presented certificates to the following nurses: Miss Griffiths, Miss Davies, Miss Peach, Miss Spivey, Miss Stonehewer, Miss Barton, Miss Carter, Miss Johnson, Miss Richards, Miss Dunsford, Miss McRae, Miss Helm, Miss Treston, Miss Hall, Miss de Chaumont, and Miss Rennie. Wigan is now taking its place as a good training school for nurses. The first course of lectures were commenced in May, 1886, and up to the present date 44 nurses have passed the final examination and obtained that certificate, and 46 the primary, and this season no less than 17 presented themselves at the primary course, of which 16 passed, and 16 presented and passed at the final. This, I think, you will say is good progress. A nurse who has faithfully discharged her duties for three years is also presented with a certificate and bonus of £5 by the Board of Management, and four of such certificates have been issued during the past year. We congratulate the above ladies on having won such excellent certificates.

### Everybody's Opinion.

[*Correspondence on all subjects is invited, but we cannot in any way be responsible for the opinions expressed by our correspondents. No communications can be entertained if the name and address of the correspondent is not given, or unless one side of the paper only be written on.*]

### THE NURSES' CO-OPERATION.

Miss R. Napper, Matron of the Surrey Convalescent Home, writes: With a view to correcting false impressions may I mention that the *National Observer*, January 30th, 1892, is under a great mistake in stating that the much-esteemed Lady Superintendent of the Nurses' Co-operation (Miss P. Hicks), was one of the founders of the scheme. She was elected from among several other applicants, as Lady Superintendent, and an excellent one she has so far proved. She has thrown herself heart and soul into the work; we value her services most highly. But the originator of the scheme is Miss M. M. Belcher; she not being able to mature the idea unaided, was advised by Mr. Henry Burdett, to whom she propounded her scheme, to apply to me to help her. For over a year we, aided by Lady Rosebery, Dr. Robert Boxall, Mr. P. Michelli, and others, were working out the details. As we did not wish to take more than five or seven-and-a-half per cent. of the nurses' earnings to pay the house rent, and salaries of the Lady Superintendent, Home Sister, and servants, also the expenses connected with the "office," money had to be advanced to meet preliminary expenses. Mr. Capel Slaughter gave us £200, Mr. Cheston, £100, and Miss Belcher, Nurse Ward, Miss Alice Napper, and myself contributed another £200. The money advanced by the nurses was under-written by two gentlemen connected with the scheme. I trust this statement of facts will correct the wide-spread misconception as to who were the original workers and starters of the Co-operation.

### THE SIR ALEXANDER MORISON PRIZES.

Dr. Urquhart writes: I enclose a copy of the circular sent to the Superintendents of the Scottish asylums, relative to the Morison prizes for meritorious attendance on the insane. One of our oldest attendants was fortunate enough in securing this distinction in 1890, and last year there was a fair muster of candidates. It must be understood that this distinction, which is only for two attendants yearly, is therefore, a prize obtainable by comparatively few attendants. It is in the power of those engaged in nursing the insane to enter for the certificate of the Medico-Psychological Association, and the very gratifying success that has followed on the inauguration of this scheme is ample proof of its necessity.

[The enclosed circular is dated from the Royal College of Physicians of Edinburgh, and says, "Will you have the goodness to send the names of eligible candidates amongst attendants on the insane of whom you have personal knowledge, with the necessary testimonials, to the Treasurer of the college before the 31st of December of this year and subsequent years." Perhaps the prizes are for Scottish asylums only; if so, will some of our readers send word?—Ed.]

### Nurses' Requisites.

We have received a sample of a silver cap or apron pin made by Mr. Bull of High Holborn. The point of the pin is fitted with a ball, so that scratching is impossible, and one has all the comfort of a safety pin combined with an elegant ornament. For nurses working in children's wards, and for those whose apron bibs have no straps, these pins should prove very useful; they are made of all lengths, and can be supplied in quantity at a reduced price. The pin is also very convenient out-of-doors for fastening nurses' cloaks; it looks best silver on a blue cloak, or silver-gilt on a brown cloak.

### The Nurses' Bookshelf.

### SWEDISH MOVEMENT.*

Already we have to acknowledge a second edition of Ostrom's small handbook on "Massage," which in its new form contains an excellent series of illustrations of Swedish movements. Of books on massage there are too many, most of them being merely compilations and repetitions, but on Swedish movements we have few works, and still fewer illustrations. Another useful addition to this edition is a complete list of the English literature on the subject—some forty books in all—so that if Ostrom is not full enough on any point, his readers can see where further information can be obtained. We can heartily recommend this handbook to nurses.

### A COMMUNITY OF WOMEN.†

The work done by the Mildmay Deaconesses is known to many of our readers, and is recalled monthly to our memories by the issue of the little grey magazine, "Service for the King." In eighteen London districts, in four English towns, in three foreign hospitals, these women carry on the good work inaugurated by the Pennefathers. Orphanages, night schools, registry for servants, hospitals, convalescent homes —and this only one out of the many communities of women. When we see about the London streets figure after figure in the uniform which marks the wearer, as "not of the world," we wonder that any misery or destitution remains to be relieved. Sisters of St. Vincent de Paul, Sisters of St. Peter, Kilburn, Deaconesses from Mildmay, Salvation Army lasses; wondrous is the work they one and all do. Here is a brief note from one of the Mildmay hospitals: "Bethnal Green.—As our report will soon be forthcoming, we will only cover space enough to say that every bed is occupied, and so are the workers too!" And here is the record from the Nursing Home, Newington Green, from which nurses are sent out to private cases: "The Nursing Home.—As usual at this season of the year the 'headquarters' of our large staff presents rather a forlorn aspect as regards numbers! But it is cheering to learn from the Superintendent that her scattered family is in good health, although many are at very trying posts, owing to epidemics which generally make their appearance in the fall of each year. Our nurses need upholding very constantly in our prayers, 'that the arms of their hands may be made strong by the hands of the mighty God of Jacob.'"

* Massage and the Swedish Movement, by Dr. Ostrom. (H. K. Lewis. Price 3s. 6d.)

† "Service for the King." (Shaw and Co. Price 2d.)

### Notes and Queries.

To Correspondents.—1. Questions or answers may be written on post-cards. 2. Advertisements in disguise are inadmissible. 3. In answering a query please quote the number. 4. A private answer can only be sent in urgent cases, and then a stamped addressed envelope must be enclosed. 5. Every communication must be accompanied by the writer's full name and address, not necessarily for publication. 6. Correspondents are requested to help their fellow nurses by answering such queries as they can.

### Answers.

*A. Nightingale.*—Your letter is written on both sides of the paper.

*F. D.*—No room for your verses. Our poets have been far too numerous lately.

*Nurse Groom.*—Two shillings received, with many thanks.

*Matron.*—Get South's "Household Surgery," published by Murray, price (we think) 2s. 6d.

*B. H.*—The Brownlow Hill certificate is considered a good one, and the hospital is fairly comfortable. You ought to be eligible for other posts if you take it, but many advertisements have lately ended, "only London-trained nurses need apply." Brownlow Hill is as good as any hospital in Liverpool.

*A Medical Nurse.*—"A Manual of General Pathology," by Joseph F. Payne, published by Smith Elder, price 12s. 6d.

### "The Doctor's Substitute."

Harry Millbanks, a very little man, and Rhoda, his wife, a very little woman, were a very young couple. They had been married three months, and had taken a very small house in Kensington for three years.

One foggy afternoon Harry came home from his office in the city complaining bitterly, but what was the exact matter of his complaint his anxious little wife could not quite make out. "What a pity, dear," she said sympathisingly, "I hope you are not going to be ill—just as we were going to celebrate your twenty-second birthday."

"And your eighteenth, the next day," Harry broke in, in a voice hoarse, but full of tender feeling.

"Are you cold, darling?" she asked.

"A little shivery, I think, dear. I felt it down my back just now."

"And the pain is here?" she said, kissing his forehead.

"Higher up; Yes, just there; but I hope I'm not going to give you a lot of trouble, Rhoda."

Rhoda smiled, with the pride of a princess, and matronly importance of a grandmother.

"You must have some hot soup," she said, "and a hot bath with mustard in it, and I'll send for the doctor."

"The doctor!" Harry exclaimed. "What nonsense, darling. Give me that book, 'The Doctor's Substitute,' that's it—no, not that—the next one—the blue book—on the other side—Yes, that's it—Thank you! This is my doctor. The idea of paying fees to old Dr. Story when I can diagnose myself in this!"

"But you might be mistaken, dear," Rhoda suggested.

"Not I—the instructions are as clear as a pike-staff. Doctors always lead to nurses, and expense—Oh, oh!"

"Poor Harry!"

"It's my head and my chest. My head's all pins, and my hest's lead."

"And the pains only began—?"

"This morning."

"You must have caught cold coming home from Mrs. Westropp's dinner party last night. It was very chilly there. That dining-room was quite draughty, and we did take such a long time over dinner."

"Oh, oh!"

"Poor Harry! where is it now?"

"It was my chest then. Ah! those pillows are beautiful. No, don't poke the fire, the room's too hot. I'm in a burning fever." Harry opened "The Doctor's Substitute" as he spoke. His wife whispered—"Look out, 'Fever,'—dear."

"Yes, 'Fever,'—F. Let me see—L, M, N.—Oh! how stupid I am. I'm quite stupid, dear!"

"Poor Harry!"

"Here's 'Ague or Intermittent Fever.'" Harry ran his eyes down the page. "My dear!" he exclaimed a minute later. "Fetch me a looking glass. Did my face become pale, and my features shrink just now? I shivered I know. That was the cold stage; and now the hot stage is coming on—'dry, burning body . . . . countenance flushed . . . tumid! Acute pain in head.' My dear, my very symptoms."

Rhoda put her hand on her husband's forehead to feel his fever. "Am I very burning, dear—tumid?" he asked anxiously.

"No, not very—at least, I don't think so."

"Oh, oh—the pain!"

"Where is it now, dear?"

"In my chest."

Rhoda took the book from her husband, and consulted it. "There's nothing about chest pains under 'Ague,' she said. It can't be that—I'll look up 'Chest.'"

"'Respiratory organs,'" Harry moaned.

"'Pneumonia!'" Rhoda exclaimed, and read on, "'generally announces itself with a chill, or a chilly feeling.' Oh, Harry, it's pneumonia!"

"Give me the book," Harry cried. He read aloud, "'Then a sensation of heat. . . Temperature rises . . . pain'—ah, pain!—'in the back and loins.' No, dear. It's not pneumonia—it's my head—I feel quite giddy!"

"Look out 'Head,' dear—H," she said. Harry turned over the pages. His fingers fixed one.

"'Hydrocephalus!'" he exclaimed. His wife caught his arm. "What a dreadful thing—you haven't got that!" she cried, beseechingly. He read, "'It's main feature is an accumulation of fluid in the central cavities of the brain.'"

"But what are the symptoms?" Rhoda asked, "Let me look?" He surrendered the book tremblingly. His wife's eager sight devoured the lines of print. "Thank goodness!" She breathed a long sigh of relief—"That dreadful thing is only a disease that children have when they are learning to walk."

"Are you quite sure, dear?" Harry moaned. "My head feels so giddy. The book!"

"Poor Harry! Let me get you some brandy. I'm sure it would be good for you." Rhoda left the book to him, and went for the brandy, confident in her own mind of its efficacy. She remembered that he always prescribed it for her; and how she hated touching it. But it would doubtless be good for him. As she was leaving the room she heard him call out, "'Cerebral Meningitis.'" What on earth could that be, she thought, and used all the more haste to return to him.

She found him sitting bolt upright in his chair, his eyes starting, and his face generally a picture of terror and misery. "I've found it out, dearest," he said, in a sad, hopeless tone of voice. "It is 'cerebral meningitis'—inflammation of the membranes of the brain—that's brain fever, I suppose. The cold shiver was nothing. The chest pains are merely sympathetic. Brain fever, my dear. How many are you? I see two heads, millions of fingers. Is that your eye, or is it the wine-glass? Oh, its brain fever right enough. 'Severe headache—intolerance of light'; the sun seems to be shining right over my head! 'Mental disquietude'—I can think of nothing. 'Unnaturally acute hearing'—I could hear you breathing all the way from the dining-room! 'Delirium'—am I delirious dear?"

"Here, drink this darling." Rhoda put the wine glass to his lips with unsteady hands, for she was very frightened. He gulped down the brandy.

"Oh, oh!" he cried in a moment or two. "My head is coming off. The ceiling is upside down. That curtain used to be red—it's green now. Rhoda, are you in the room?"

She had snatched up "The Doctor's Substitute."

"I'm here," she answered. "You must go to bed." And closing the book, she repeated to herself: "Dark room—send for the doctor."

"Does the book say go to bed, dear?" Harry asked.

"Very well, I'll go. Help me!"

Rhoda darkened the bed-room, put a cold bandage upon her husband's head, and sent for Dr. Story. The minutes passed like hours. She met the doctor at the door, and whispered to him that she was afraid her husband had brain fever. The doctor looked serious. "You've done quite right," he said, noticing the darkened room. He crossed the carpet noiselessly to the patient's bedside, felt his pulse, pressed his eyes, and asked if the pressure hurt him, made various other enquiries, especially as to the patient's mode of living during the past few days. Rhoda told him about the dinner party and other similar items. His brow cleared, and his mouth relaxed somewhat its resolute, straight line.

"It's a slight attack of indigestion!" he said at length. "I'll write a prescription."

And while he did so Harry and his wife indulged in something very like a mutual embrace.

## Figures and Tables

**Fig. 1. Fig. 2. f1:**
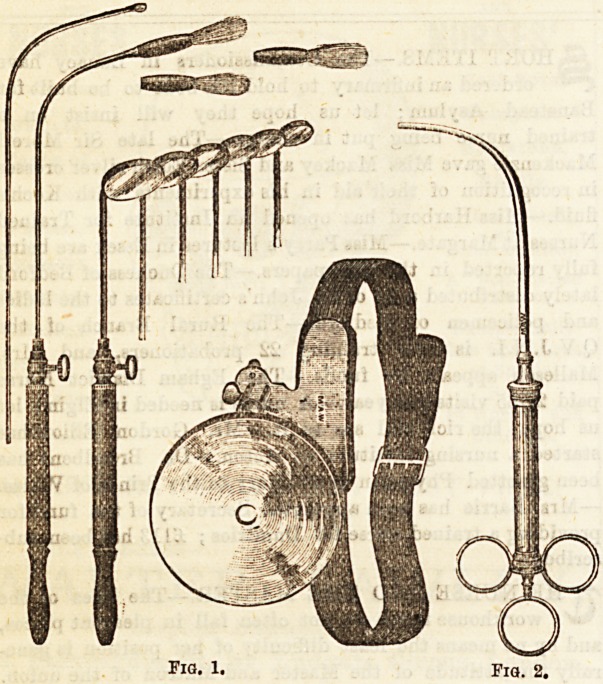


**Fig 3. f2:**
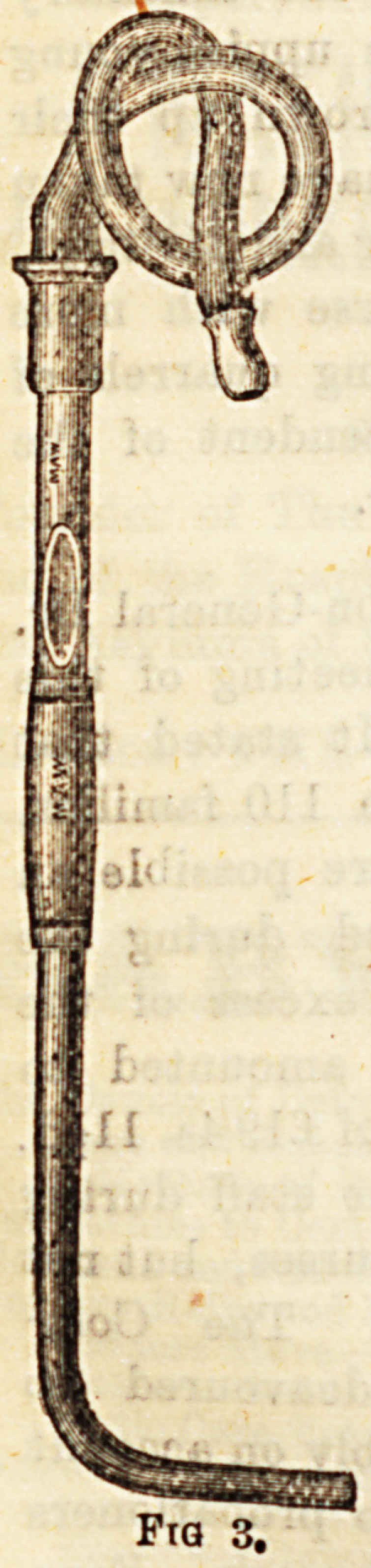


**Fig. 4. f3:**
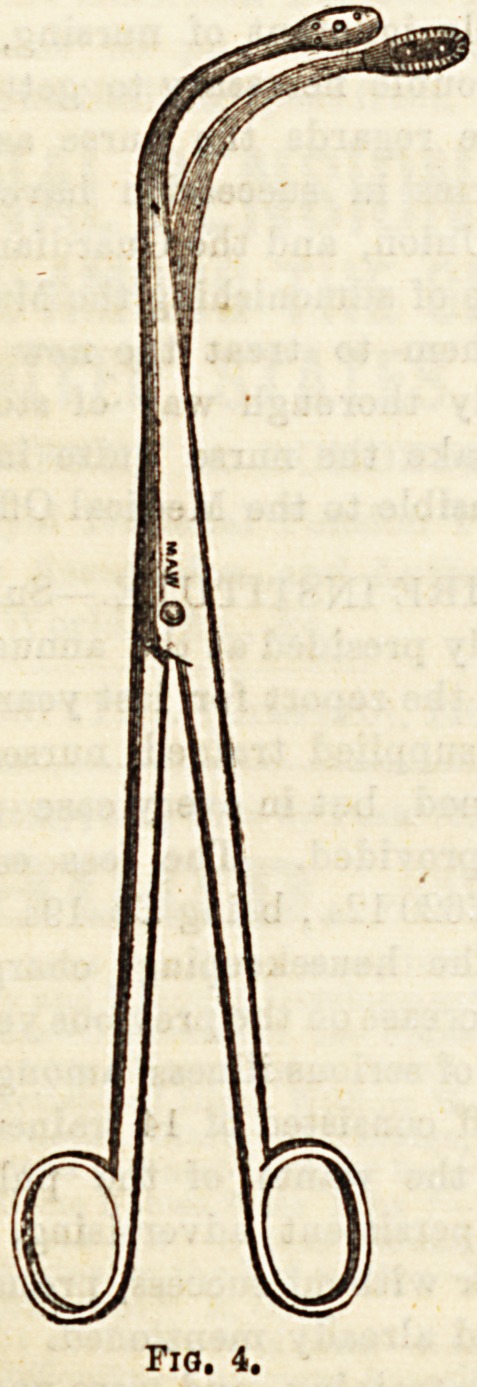


**Fig. 5. f4:**
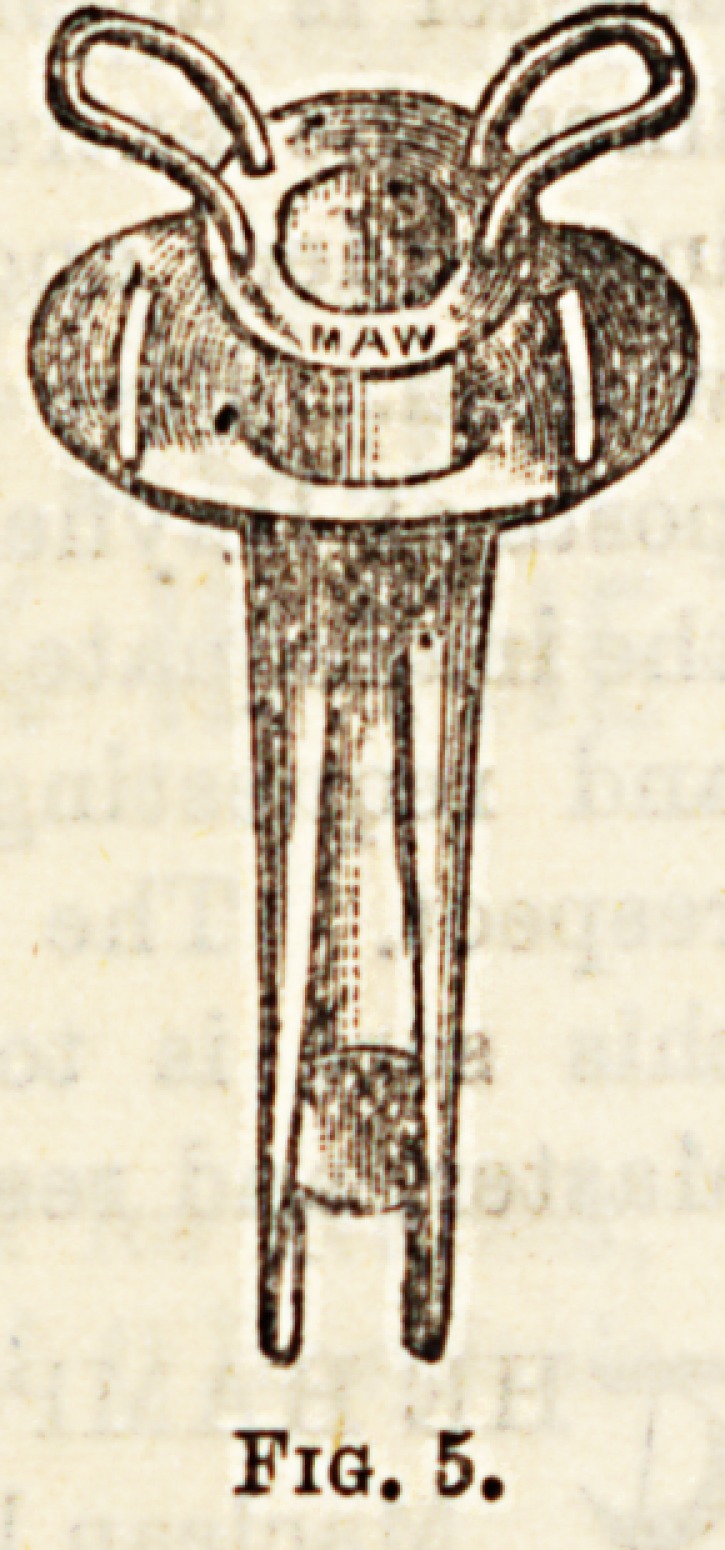


**Fig. 6a. f5:**
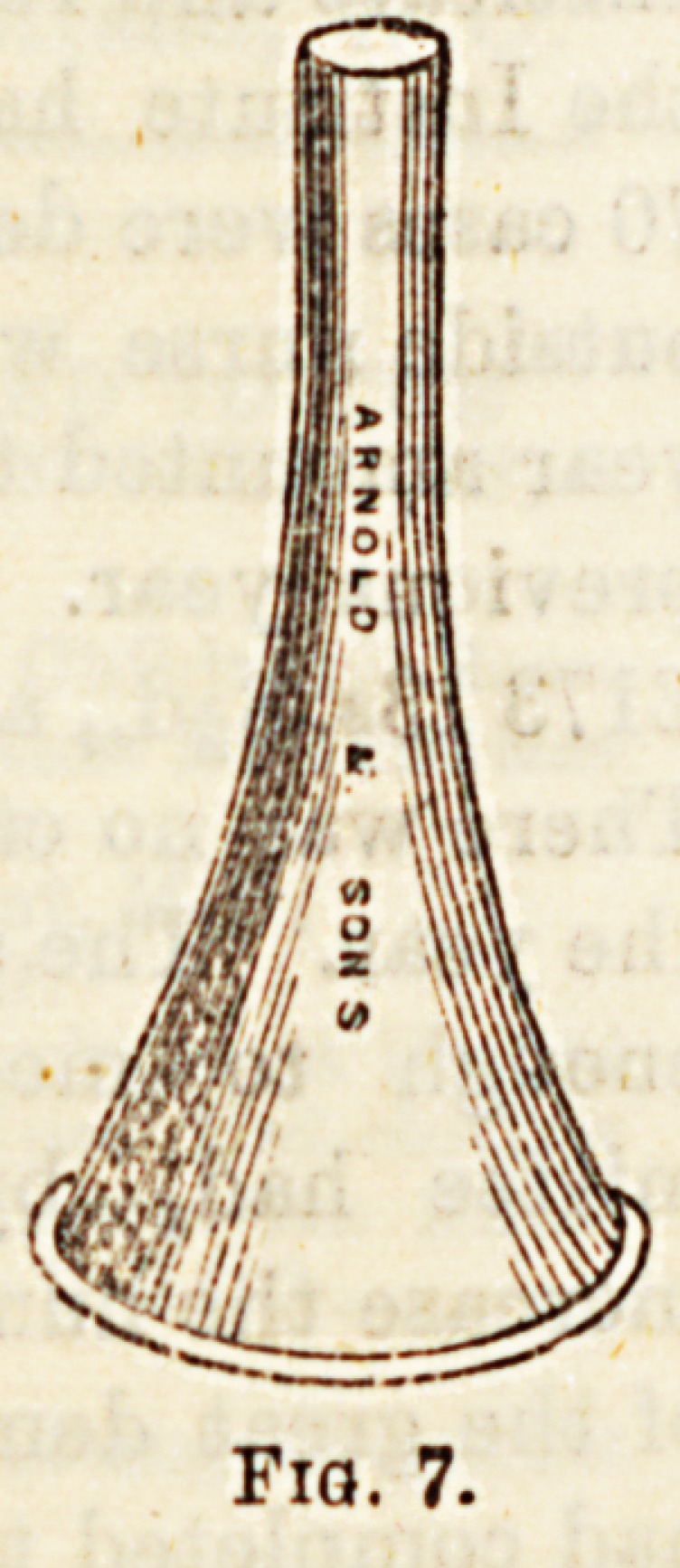


**Fig. 7. f6:**
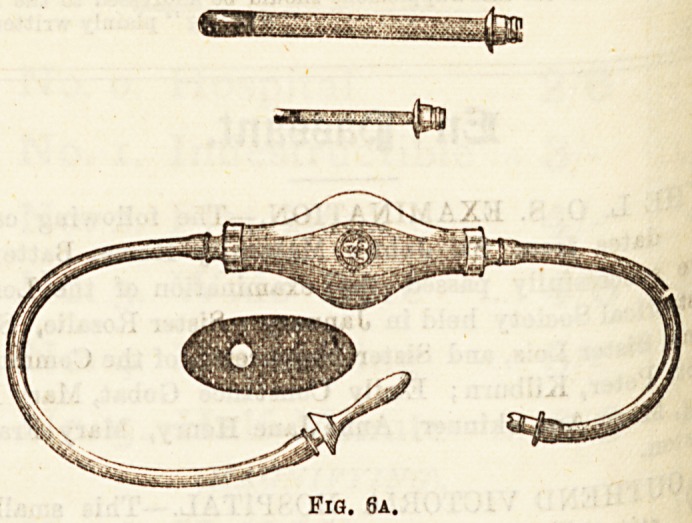


**Fig. 8. f7:**
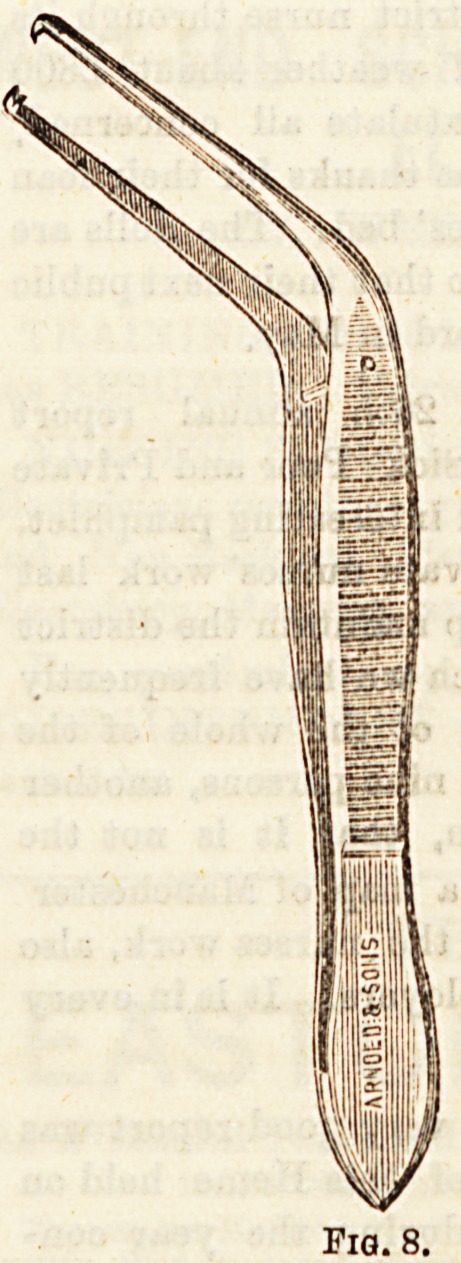


**Fig. 9. f8:**
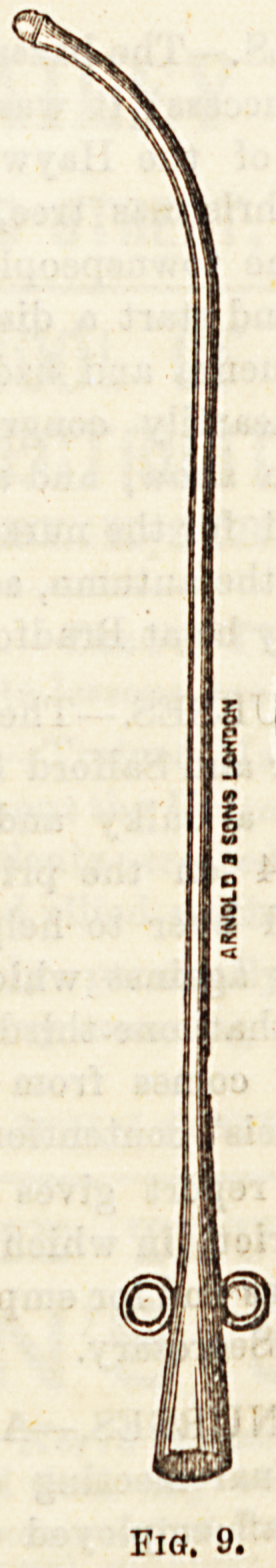


**Fig. 10. f9:**
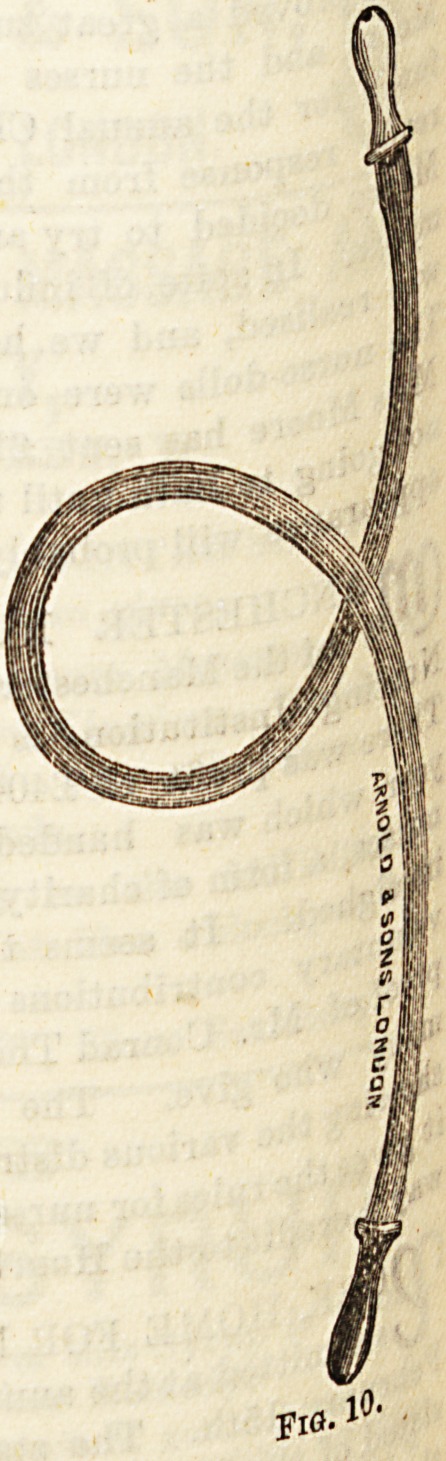


**Fig. 11. f10:**
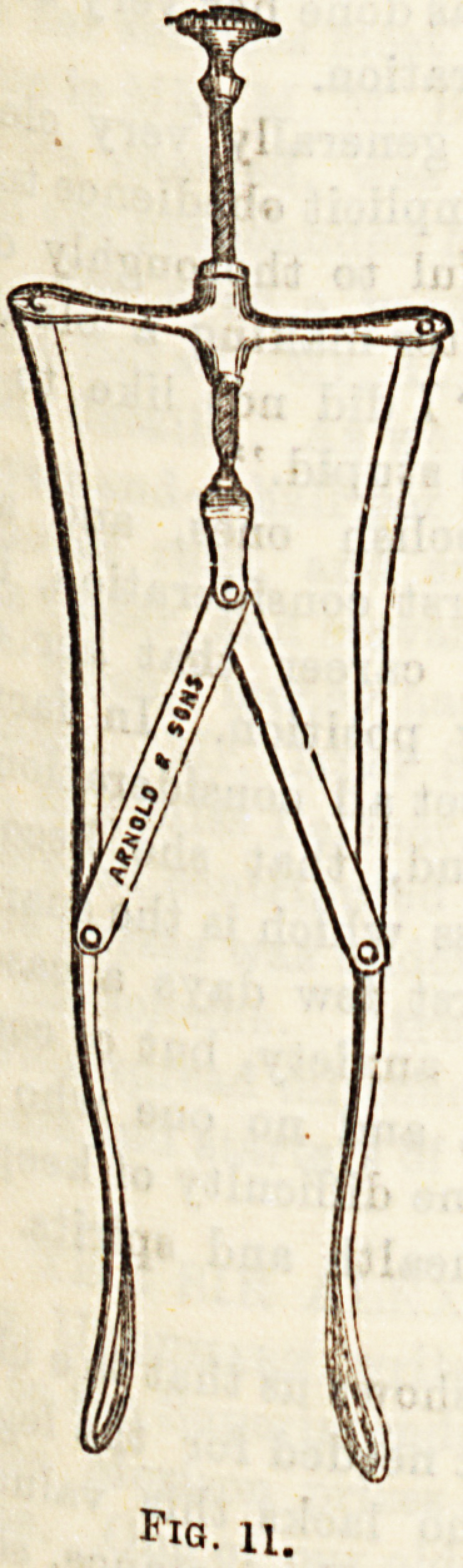


**Fig. 12. f11:**
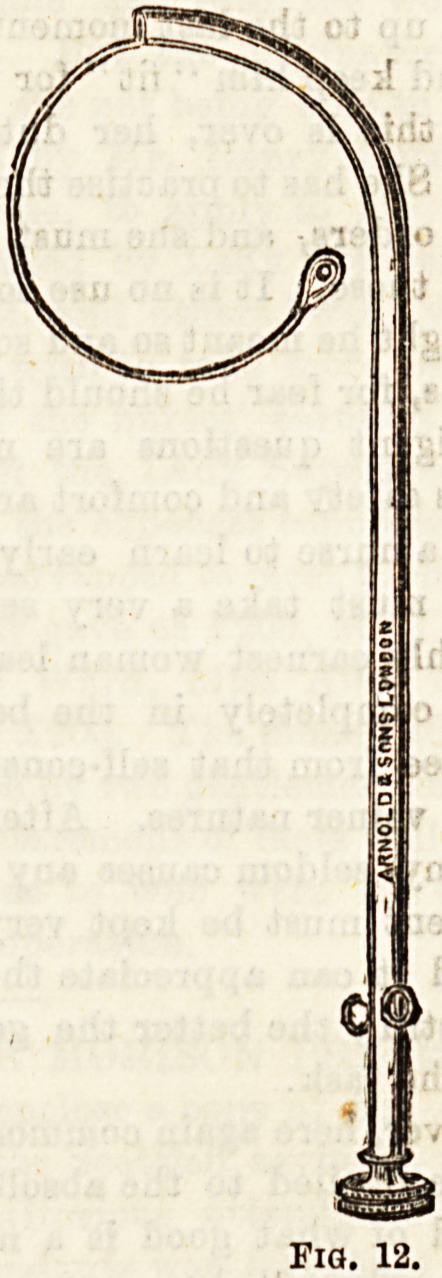


**Figure f12:**